# Serum L-selectin levels as predictive markers for chronic major depressive disorder progression

**DOI:** 10.1186/s12991-024-00522-0

**Published:** 2024-10-16

**Authors:** Yeeun Yun, Sora Mun, Seungyeon Lee, Hee-Gyoo Kang, Jiyeong Lee

**Affiliations:** 1 Department of Biomedical Laboratory Science, Graduate School, Eulji University, Gyeonggi, Republic of Korea; 2 Department of Biomedical Laboratory Science, College of Health Science, Eulji University, Gyeonggi, Republic of Korea; 3 Department of Senior Healthcare, Graduate School, Eulji University, Gyeonggi, Republic of Korea

**Keywords:** Major Depressive Disorder (MDD), Biomarkers, Serum proteomics, L-selectin, Chronic depression, Inflammatory response mechanisms

## Abstract

**Background:**

Major depressive disorder (MDD) exhibits a recurrence rate of up to 70%. Frequent recurrence can lead to chronic depression, which has considerable personal and societal consequences. This study aims to identify a serum protein biomarker to predict MDD recurrence and progression to chronicity.

**Methods:**

Serum samples from the MDD with single episode group (MDD-S), MDD with recurrence group (MDD-R), and a healthy control group were collected. Non-targeted analysis of the serum proteome was conducted using liquid chromatography–tandem mass spectrometry. Statistically significant common proteins when comparing the three groups were chosen. The selected marker candidates were subsequently validated through multiple response monitoring (MRM), incorporating a healthy control, MDD-S, MDD-R(2) (two episodes), and MDD-R(> 2) (more than two episodes) groups.

**Results:**

L-selectin levels showed an upward trend in the MDD-R group compared to the healthy control and MDD-S groups. MRM validation revealed a decreased tendency for L-selectin in the MDD-R(> 2) group, indicative of a chronic state, versus the healthy control and MDD-S groups. The receiver operating characteristic analysis highlighted L-selectin as the chosen biomarker due to its classification efficacy for the MDD-R(> 2) group.

**Conclusion:**

L-selectin emerged as a predictive biomarker for MDD recurrence and its potential evolution into chronic depression. This marker offers insights into changes in leukocyte-mediated inflammatory responses characteristic of chronic depression. Consequently, it may forecast the transition from acute to chronic inflammation in depressive patients.

## Background

According to the WHO, depression affects approximately 5% of adults globally [1]. An initial occurrence is termed Major Depressive Disorder with a single episode (MDD-S), and a second occurrence is labeled Major Depressive Disorder with recurrence (MDD-R) [2]. Recurrence rates of depression are substantial, with studies indicating 50–90% rates [3, 4].

When depression recurs, it often becomes chronic. Following a recurrence, there is a 50% likelihood of a third episode. After this third instance, the probability of a subsequent episode exceeds 70%. A third episode establishes the 'chronic' classification, prompting considerations for lifelong antidepressant use [5, 6]. Chronic depression escalates the risks of suicide attempts, substance abuse, and anxiety disorders. Extended antidepressant use can result in tachyphylaxis, affecting 25–50% of patients [7]. Despite this, predicting relapse for MDD-S patients remains elusive [8]. Thus, biomarkers for recurrence prediction could be invaluable in shaping.

The exact physiological pathway for depression remains unidentified [9]. Currently, depression diagnosis relies on subjective assessments from both physicians and patients. If a biomarker based on the pathophysiological state of depression were discovered, it would enable objective diagnosis and monitoring of depression recurrence. Recent research on depression biomarkers has revealed numerous associations with biomolecules involved in immune responses, such as inflammation [10]. Meta-analyses and systematic reviews have demonstrated significant alterations in the levels of IL-1β, IL-2, IL-4, IL-8, soluble IL-6 receptor (sIL-6R), IL-5, CCL3, IL-17A, and TGF-β1 in individuals with depression [10]. Biomarkers related to symptomatic characteristics of depression, including suicidal behavior—a marker of chronic depression—have been found to be associated with inflammation and immune response. Studies have shown that apolipoprotein A-IV-mediated inflammation plays a role in clinical depression and may induce neurological changes in the brain [11]. Moreover, feelings of hopelessness and emotional temperament have been identified as important predictors of suicide risk in MDD patients [12]. In MDD and suicide-related behavior, inflammatory cytokines such as interleukin (IL)-1, IL-6, IL-17, tumor necrosis factor-α (TNF-α), and IL-4 trigger the secretion of various anti-inflammatory cytokines, including IL-10, IL-13, and transforming growth factor-β (TGF-β) [13]. Additionally, inflammatory cytokines stimulate kynurenic acid production in the tryptophan-kynurenine pathway, which has been linked to depression [14]. The activation of the kynurenine pathway by immune responses in patients with depression, which has been linked to suicidal behavior [15], suggests that the risk of relapse in chronic depression is associated with immune responses such as inflammation. However, biomarkers for screening the risk of relapse in chronic depression have not yet been established.

Therefore, this study aimed to identify screening biomarkers for the recurrence of depression with a risk of chronicity, utilizing proteomics techniques. Proteins, central to organism function, exhibit varied expression during disease onset [16]. Proteomic approaches offer insight into altered protein profiles based on disease state, aiding in disease biomarker discovery via blood samples [17]. Unraveling proteomic biomarkers for diseases like MDD, where molecular mechanisms are vague, can pave the way for understanding MDD’s intricate molecular mechanisms [18–21]. This study seeks to discern distinct protein profiles between MDD-S, MDD-R, and healthy controls by comparing their serum protein signatures. Subsequently, validation with chronic depression patients will be pursued. These biomarkers hold potential for preempting relapse before it exacerbates into chronic depression.

## Materials and methods

### Subjects

This study assessed patients with MDD (single, recurrent) from Eulji University Hospital who were diagnosed by a psychiatrist. In the discovery set, there were 27 HC (healthy controls), 24 MDD-S(Major Depressive Disorder with a single episode), and 20 MDD-R(Major Depressive Disorder with recurrence). The validation set comprised a larger number of participants than the discovery set. For validation, patients were categorized based on the number of episodes: 66 HC, 43 MDD-S, 28 MDD-R(2) (major depressive disorder patients with 2 episodes), and 11 MDD-R(> 2) (major depressive disorder patients with more than 2 episodes). Detailed data, including the Hamilton depression rating scale (HAMD) scores can be found in Table 1. Additionally, MDD-R(> 2) in our sample comprised patients with episodes lasting at least 2 years, according to the diagnostic criteria of DSM-IV [5]. As recurrence can occur in 90% of patients who have had more than two episodes of depression, it can serve as an important time point for predicting recurrence as well as chronicity [5, 6]. Subjects in the discovery set underwent serum protein profiling using liquid chromatography–tandem mass spectrometry (LC–MS/MS). The selected candidate biomarkers were then validated using multiple reaction monitoring (MRM) analysis, which allows for the absolute quantification of these biomarkers. MRM serves as a method to validate the identified candidate biomarkers.

**Table 1:** Demographic details of the participants

Sample set		Discovery set			Validation set			
		HC^c^ (n = 27)	MDD-S^d^ (n = 24)	MDD-R^e^ (n = 20)	HC (n = 66)	MDD-S (n = 43)	MDD-R(2)^f^ (n = 28)	MDD-R(> 2)^g^ (n = 11)
Sex	Male/female	6/21	7/17	6/14	13/53	10/33	8/20	3/8
Age	Mean ± SD^b^	56 ± 11	43 ± 12	49 ± 17	61 ± 10	47 ± 18	52 ± 19	59 ± 17
HAMD-17^a^	Mean ± SD^b^		21 ± 6	19 ± 4		21 ± 6	21 ± 5	19 ± 7
Drug treatment								
+	n		12	11		22	11	10
−	n		12	9		21	17	1

^a^HAMD-17: Hamilton depression rating scale, ^b^SD: Standard deviation, ^c^HC: Healthy controls, ^d^MDD-S: Major depressive disorder patients with single episode, ^e^MDD-R: Major depressive disorder patients with recurrence, ^f^MDD-R(2): Major depressive disorder patients with 2 episodes, ^g^MDD-R(> 2): Major depressive disorder patients with more than 2 episodes

### Serum sample collection and processing for mass spectrometry analysis

Serum samples from patients diagnosed with MDD, both single episodes and multiple episodes, were collected at Eulji University Hospital. Blood samples were drawn into anticoagulant-free vacutainers and incubated at 24 °C for 2 h before being centrifuged at 4000 ×*g* for 5 min. A multiple-affinity removal system using a liquid chromatography (LC) column (human 6-HC, 4.6 × 50 mm; Agilent Technologies, Santa Clara, CA, USA) was used to deplete six highly abundant proteins from the human serum before tryptic digestion. In essence, the samples were loaded onto the column to isolate low-abundance proteins for subsequent analysis. To concentrate proteins in the samples, a Nanosep Centrifugal Device equipped with an Omega™ Membrane 3 K (Pall Corporation, Port Washington, NY, USA) was employed. Sample preparation for serum proteome and mass spectrometry methods performed in this study were based on previously established protocols [11, 22–25]. To determine protein concentration, we utilized a BCA Protein Assay Kit (Thermo Fisher Scientific, Cleveland, OH, USA) following the manufacturer’s guidelines. Samples were prepared to achieve a protein concentration of 100 μg. For reduction, the samples were transformed into peptides by treating them with 5 mM Tris (2-carboxyethyl) phosphine (Pierce, Rockford, IL, USA) at 37 °C. After centrifuging at 400 rpm for 30 min, the samples were alkylated using 15 mM iodoacetamide (Sigma-Aldrich, St. Louis, MO, USA) at 25 °C for 1 h in the dark. Subsequently, serum proteins were digested into peptides using mass spectrometry (MS)-grade trypsin gold (Promega, Madison, WI, USA) and incubated overnight at 37 °C. Finally, the resulting peptides were desalted using a C18 cartridge (Waters, Milford, MA, USA), and the purified samples were dried using a vacuum dryer (ScanVac, LaboGene, Lynge, Denmark).

### Protein fractionation of samples and LC–MS/MS analysis

Pooled serum samples were divided into 12 fractions using the 3100 OFFGEL Low Res Kit (pH 3–10; Agilent Technologies) as per the manufacturer’s guidelines. These fractions were then purified with a C18 Macro spin column (Harvard Apparatus, Holliston, MA, USA) and dried via a vacuum dryer (Scan Vac, LaboGene). Each of the 12 fractions was loaded onto an Eksigent nanoLC 400 system equipped with cHiPLC^®^ (AB Sciex, Concord, ON, Canada). For both individual and relative sample analysis, a TripleTOF 5600 mass spectrometer (AB Sciex) was used, leveraging the sequential window acquisition of all theoretical mass spectra (SWATH) acquisition, which is a data-independent acquisition (DIA) technique. Samples, with a concentration of 1 µg/µL, were introduced into an Eksigent ChromXP nanoLC trap column (350 µm i.d. × 0.5 mm, ChromXP C18 3 µm) at a flow rate of 5000 nL/min. Elution from the Eksigent ChromXP nanoLC column (75 µm i.d. × 15 cm) was carried out at a flow rate of 300 nL/min over a span of 95 min. During this period, the mobile phase B buffer was progressively increased in the column (from 5 to 90%) using the following gradient: 0 min at 5% mobile phase B, 10.5 min at 40%, 80 min at 90%, and returning to 5% in 95 min.

### Synthesis and purification of label-free standard peptides and quantification using MRM analysis

For absolute quantification, peptides were synthesized by Peptron Co. (Yousung, Korea) adhering to strict criteria: absence of miscleaved sites, peptides remaining unmodified, exclusion of Met, and peptide lengths ranging from 7 to 15 residues to ensure a low false discovery rate (< 1%). After synthesis, peptide standards were synthesized through two-fold serial dilutions starting at 1 μM in HPLC-grade water, following the manufacturer’s instructions. Using the Skyline library, MRM Q1/Q3 ion values were obtained, and a transition test was performed to determine optimal CE and BP values. Samples were separated using an SCIEX ExionLC equibbed with a C18 column (ACQUITY UPLC BEH C18 Column [130 Å, 1.7 µm, 2.1 mm × 150 mm]) and an accompanying ACQUITY UPLC BEH C18 VanGuard Pre-column [130 Å, 1.7 µm, 2.1 mm × 5 mm]. Following separation, the QTRAP 5500 (AB Sciex) was utilized to analyze samples from MDD-S, MDD-R(2), MDD-R(> 2), and healthy subjects. Samples were loaded onto the LC column using a gradient from 5 to 90% of mobile phase B over 30 min. The gradient progression for mobile phase B was as follows: 1 min at 5%, 50 min at 40%, from 21 to 25 min at 90%, and from 25.5 to 30 min returning to 5%. Mobile phase B consisted of 0.1% formic acid in HPLC-grade acetonitrile; mobile phase A was 0.1% formic acid in HPLC-grade water. MRM analysis source parameters included a curtain gas at 30 psi, low collision gas, ion spray voltage at 5500 V, a temperature at 400 °C, ion source gas 1 at 40 psi, and ion source gas 2 at 60 psi.

### Statistical analysis

Data processing involved the use of PeakView for peak detection and MarkerView software for total sum normalization and t-tests. Proteins with a p-value less than 0.05 were subjected to further analysis. MRM analysis results were evaluated using MultiQuant software to determine the absolute concentration of the analyte. Outliers were identified, and all data were subsequently cleaned. The statistical tests applied included the Mann–Whitney test, unpaired t-test, and later, the receiver operating characteristic (ROC) test. All statistical analyses were conducted using GraphPad Prism software version 5.0 (La Jolla, CA, USA).

## Results

### Comparison of protein identification in pooled and individual serum samples

**Fig. 1:**
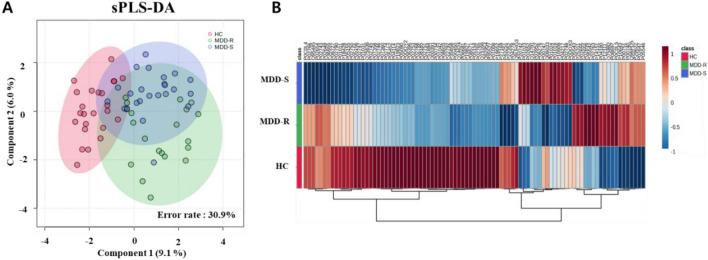
Proteomic analysis of proteins from the healthy control group and the major depressive disorder patient group. **A** sPLS-DA of 89 differentially expressed proteins. Healthy controls (HC) are represented by red dots and area, MDD-S (major depressive disorder—single episode) by blue dots and area, and MDD-R (major depressive disorder—recurrent episodes) by green dots and area. **B** Heatmap of the 89 differentially expressed proteins. The horizontal axis denotes the 89 differentially expressed proteins, while the vertical axis represents the average relative concentration of proteins for each group: HC in red, MDD-R in green, and MDD-S in blue. For individual proteins, a higher presence is indicated in red and a lower presence in blue

An ion library was constructed using IDA data from pooled samples. Subsequently, 89 proteins were identified by aligning the SWATH data of individual samples with the ion library. After conducting sPLS-DA on these 89 proteins, the components were determined to be 9.1% and 6.0%, with an error rate of 30.9%. The groups compared did not fully overlap within the confidence interval, making classification between them challenging, as depicted in Fig. 1A. A heatmap analysis further confirmed the average expression level of the 89 proteins in each group (Fig. 1B).

### Protein profiling for recurrent MDD discovery

**Fig. 2:**
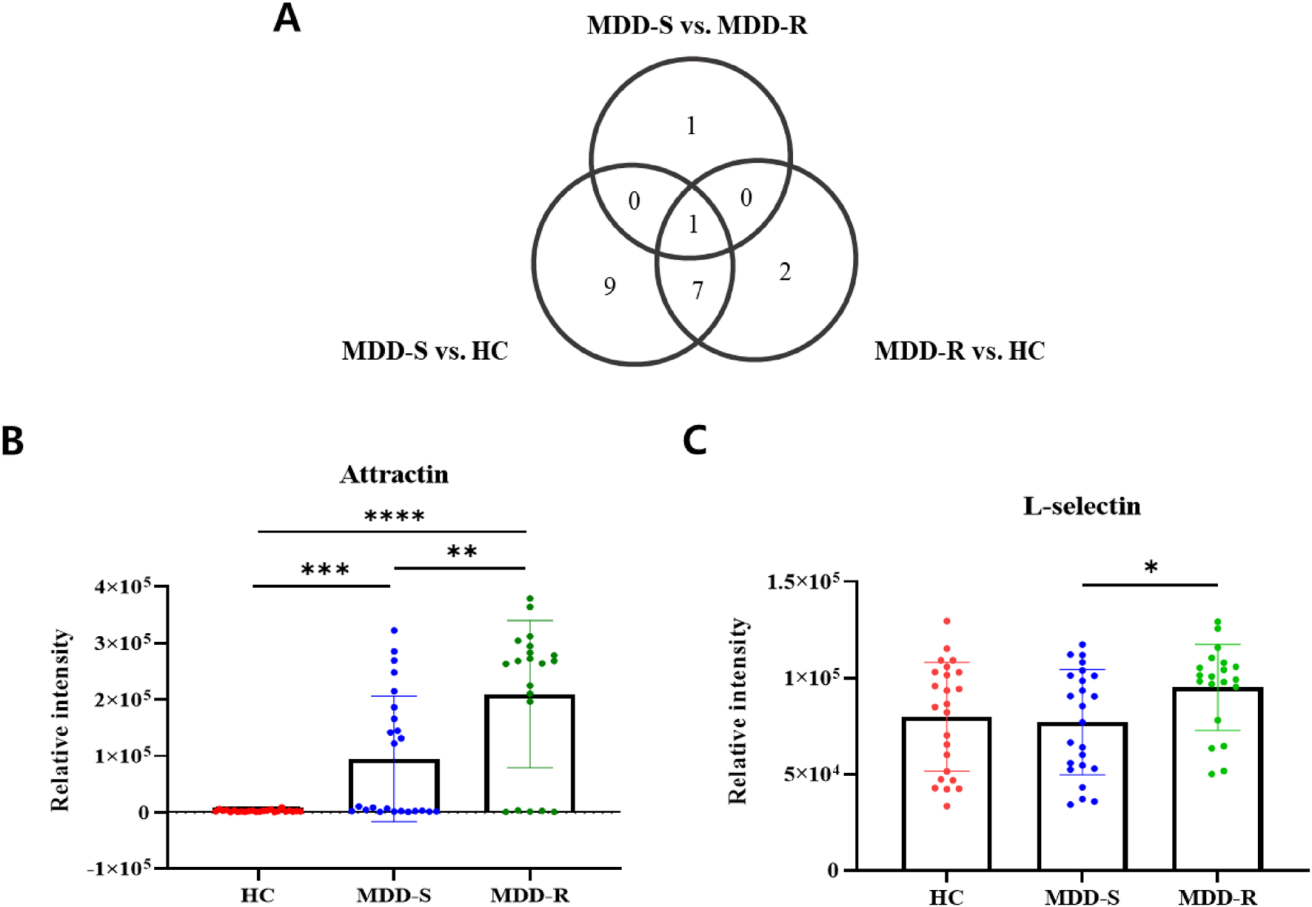
Differentially expressed proteins across groups. **A** Venn diagram illustrating differentially expressed proteins by comparison combination. **B, C** Comparison of relative quantities of selected marker candidates among groups. For intergroup comparisons, either the unpaired t-test or the Mann–Whitney test was used. The values displayed on the graph represent the mean ± SD. Significance levels are denoted as *P < 0.05, **P < 0.01, ***P < 0.001, and ****P < 0.0001

By comparing the MDD-S, MDD-R, and HC groups, we identified differentially expressed proteins meeting criteria of a p-value ≤ 0.05 and a fold change ≥ 1.2. The results were visualized using a Venn diagram (Fig. 2A). Between MDD-S and MDD-R, two proteins differed. In comparison between MDD-R and HC, there were 10 distinct proteins and 17 between MDD-S and HC. We focused on the differing proteins between MDD-S and MDD-R as potential candidate markers. Specifically, Attractin levels progressively increased from HC to MDD-S and then to MDD-R. This increase was statistically significant across all group comparisons. Notably, Attractin may serve as a marker to distinguish not only between healthy individuals and those with MDD but also between MDD-S and MDD-R cases (Fig. 2b). L-selectin was elevated in MDD-R compared to both HC and MDD-S, although this difference was not always significant. Nevertheless, due to its significant variance between MDD-S and MDD-R, L-selectin was also chosen as a candidate marker (Fig. 2C). Details on the selected candidates are provided in Table 2.

**Table 2:** List of potential candidate proteins

No.	Uniprot ID	Gene name	Protein name	*p*-value	Fold change (MDD-R/MDD-S)
1	O75882	ATRN	Attractin	0.0036	2.21
2	P14151	SELL	L-selectin	0.0198	1.24

MDD-R: Major depressive disorder patients with recurrence; MDD-S: Major depressive disorder patients with single episode

### Validation of potential biomarkers

Table 3 lists the peptides and MRM parameters chosen for protein quantification. For validation via MRM, patients with MDD-R were categorized into two groups: those with MDD who had experienced 2 episodes (MDD-R(2)) and those with more than 2 episodes (MDD-R(> 2)). This categorization aimed to assess the marker’s significance not only in recurrence but also in chronic recurrence.

**Table 3:** MRM transitions used for validating candidate proteins

No.	Uniprot ID	Gene name	Protein name	Peptide sequence	Q1/Q3	Ion type	DP	CE
1	O75882	ATRN	Attractin	LTLTPWVGLR	578.3479/828.4726	2y7	80	25
2	P14151	SELL	L-selectin	SYYWIGIR	529.2769/807.4512	2y6	80	25

DP: Declustering potential, CE: Collision energy

**Fig. 3:**
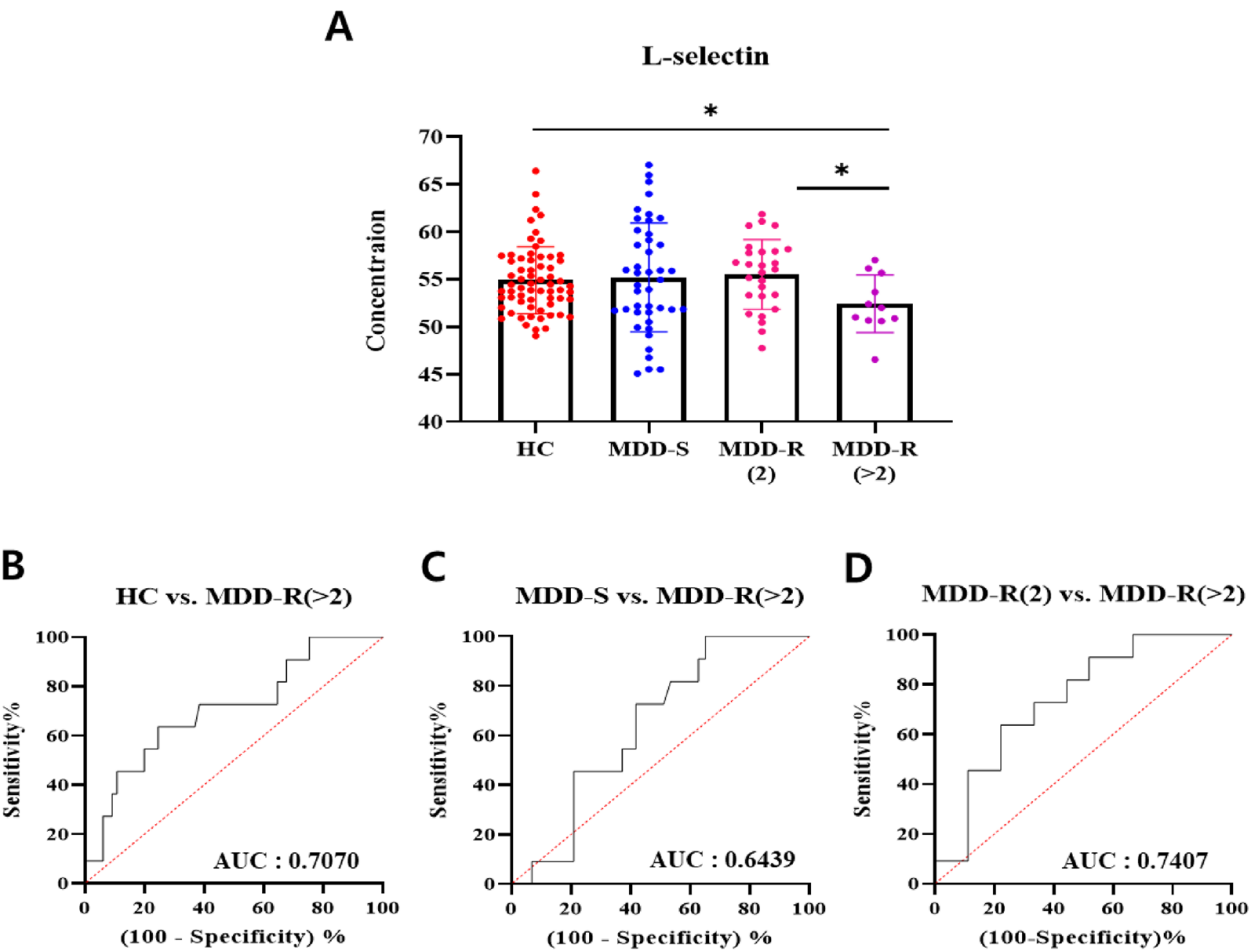
MRM quantitative comparison of L-selectin across groups. **A** Scatter plots depict the MRM quantitative values for each group. Comparisons between groups were made using the unpaired t-test or the Mann–Whitney test, with values in the graph presented as mean ± SD. *P < 0.05. **B**–**D** ROC (Receiver Operating Characteristic) curves comparing L-selectin levels between the Healthy control and recurrent groups. **B** ROC curve for HC vs. MDD-R(> 2), **C** ROC curve for MDD-S vs. MDD-R(> 2), and **D** ROC curve for MDD-R(2) vs. MDD-R(> 2). The area under the curve (AUC) for each ROC is displayed in the bottom right corner of the respective graph

The results revealed that Attractin did not show a significant difference across all group comparisons. Conversely, while L-selectin did not show a significant difference between MDD-S and MDD-R(2), it was significantly different in the MDD-R(> 2) group (considered chronic) when compared to both HC and MDD-R(2). Although there was no significant difference between MDD-S and MDD-R(> 2), a declining trend was observed (Fig. 3A). Furthermore, the ROC analysis for L-selectin betwen HC and MDD-R(> 2) resulted in an AUC of 0.7070 (Fig. 3bB). The AUC values from the ROC analyses between MDD-S and MDD-R(> 2) and between MDD-R(2) and MDD-R(> 2) were 0.6439 (Fig. 3C) and 0.70407 (Fig. 3D), respectively. These ROC outcomes suggest that chronic depression can be differentiated from patients with MDD-S or MDD-R(2). Given these findings, L-selectin was chosen as a predictive biomarker for recurrence, particularly in cases suspected of being chronic.

## Discussion

This study aimed to identify serum protein biomarkers that could predict relapse and progression to chronicity in MDD. The results revealed that L-selectin levels were statistically significantly reduced in MDD-R(> 2), which represents chronic cases, compared to the control group, MDD-S, and MDD-R. Therefore, L-selectin shows potential as a potential predictive biomarker for MDD relapse and chronic depression.

L-selectin is found on the surface of all leukocyte types and plays a role in leukocyte migration or chemotaxis [26]. It is rapidly shed within minutes after activation by various cytokines and chemotactic factors [27] and can remain bound to its ligand or be released into the plasma fraction as sL-selectin [27]. The release of soluble L-selectin from leukocytes modulates their activity and the inflammatory response [26]. Hypotheses regarding the relationship between depression and inflammation have been made in several papers [28, 29]. For instance, studies linking neutrophils and inflammation suggest that the L-selectin-dependent pathway plays an important role in neutrophil migration and adhesion. [30]. Cell adhesion molecules may link peripheral inflammation and neuroinflammation in individuals with severe psychiatric disorders by enhancing inflammatory and immune-mediated responses and transducing blood–brain barrier-mediated signaling [31]. L-selectin is associated with MDD as well as mental diseases such as Alzheimer’s disease [32] and schizophrenia [33]. In particular, L-selectin is reported to be increased in schizophrenia [33] and MDD [34].

In this study, we observed a decrease in L-selectin levels in the blood of chronically depressed patients with MDD-R(> 2) compared to MDD-S and MDD-R(2) patients. The tendency for decreased L-selectin in chronic states has also been reported in other diseases [35]. This reduction is indicative of changes in chronic depression distinct from general depression (MDD-S or MDD-R(2)). We postulate that this reflects alterations in the leukocyte-mediated inflammatory response regulated by L-selectin due to the chronic nature of MDD-R(> 2) depression. A significant proportion of depression patients experience recurrent episodes, many of which are resistant to treatment [36]. Treatment-resistant depression patients are characterized by elevated inflammatory proteins, with increased interleukin 6 and 8 linked to poorer treatment outcomes [37]. Interleukin 6, in particular, is a pro-inflammatory cytokine that is significantly elevated in depressed patients [38–41], and is known to regulate L-selectin expression [42]. Furthermore, reduced L-selectin expression in polymorphonuclear leukocytes (PMN) in environments with increased interleukin 6, suggesting that IL-6 diminishes L-selectin levels in circulating PMNs by promoting the release of PMNs with low L-selectin expression from the bone marrow [42].

IL-6 is pivotal in the acute phase response. Persistent activity of pro-inflammatory cytokines like IL-6 can transition acute inflammation to chronic inflammation involving immune responses. In chronic inflammation, IL-6 promotes monocyte accumulation at injury sites through sustained MCP-1 secretion on T cells, angiogenesis, and antiapoptotic functions [43]. This escalates serum IL-6 levels, potentially initiating the amplification phase of chronic inflammatory proliferation [44]. Similarly, patients with chronic depression, especially those with multiple episodes, are continually exposed to IL-6. It is plausible that the observed L-selectin decrease in MDD-R(> 2) patients results from sustained IL-6 exposure, leading to the release of PMNs with low L-selectin expression. In conclusion, the reduction in L-selectin in patients with treatment-resistant multiple episodes and chronic depression arises from continuous exposure to IL-6 and the transportation of PMNs with low L-selectin expression from the bone marrow. These circulating PMNs, which play a role in releasing sL-selectin into the blood, may have contributed to the sL-selectin presence during the inflammation process. L-selectin serves as a valuable marker for monitoring leukocyte-mediated inflammatory response changes seen in chronic depression. This marker helps identify the shift from acute to chronic inflammation in depressed patients, potentially aiding in predicting recurrence.

Lastly, this study has some limitations in controlling confounding variables such as sex, age, medication, and comorbidities. First, in the case of age, one-way ANOVA was performed, and confounding variables in HC and MDD-S were not controlled in the discovery and validation sets. Related information was confirmed through Scheffe’s post hoc test. In the case of drugs, chi-square analysis was performed. Therefore, because HC did not take medication, confounding variables were not controlled. In addition, for MDD-R(> 2), samples without drug exposure could not be obtained. Comorbidities were also analyzed using chi-square test, and confounding variables were not controlled. However, sex was confirmed to be a control variable through chi-square analysis. The results for which confounding variables were not controlled in this study require additional verification in the future. Additionally, future studies will enable expanded research into the mechanisms involved by further verifying L-selectin-related immune and inflammatory response pathway proteins.

## Conclusion

In MDD-R(> 2), categorized as chronic, there was a notable decrease in L-selectin levels. This suggests that IL-6 may lead to reduced L-selectin levels in the context of depression. Information on changes in L-selectin suggests that the recurrence and chronicity of depression are linked to immune responses, such as inflammation. The immune network related to L-seleectin may enhance our understanding of the pathophysiological mechanisms underlying the recurrence and chronicity of depression. Furthermore, biomarkers like L-selectin could facilitate the screening of patients at high risk for chronicity, thereby enabling targeted treatments for relapse and chronic depression. This study offers crucial insights into the chronic nature of depression.

## Data Availability

No datasets were generated or analysed during the current study.

## Abbreviations

HC: Healthy controls
HAMD: Hamilton depression rating scale
LC–MS/MS: Liquid chromatography–tandem mass spectrometry
MDD: Major depressive disorder
MDD-S: Major depressive disorder with a single episode
MDD-R: Major depressive disorder with recurrence
MDD-R(2): Major depressive disorder patients with 2 episodes
MDD-R(> 2): Major depressive disorder patients with more than 2 episodes
MRM: Multiple response monitoring
PMNs: Polymorphonuclear leukocytes
ROC: Receiver operating characteristic
