# Correction: Serum L-selectin levels as predictive markers for chronic major depressive disorder progression

**DOI:** 10.1186/s12991-025-00594-6

**Published:** 2025-08-22

**Authors:** Yeeun Yun, Sora Mun, Seungyeon Lee, Hee-Gyoo Kang, Jiyeong Lee

**Affiliations:** 1 Department of Biomedical Laboratory Science, Graduate School, Eulji University, Gyeonggi, Republic of Korea; 2 Department of Biomedical Laboratory Science, College of Health Science, Eulji University, Gyeonggi, Republic of Korea; 3 Department of Senior Healthcare, Graduate School, Eulji University, Gyeonggi, Republic of Korea

**Correction to: Ann Gen Psychiatry 23, 37 (2024)**

10.1186/s12991-024-00522-0

In the original publication of this article [1], there is a typo in “Ethics approval and consent to participate” section. The IRB approval number was incorrectly given as “EU2*2*-46” but should have been “EU2*3*-46”. The incorrect and correct Ethics Declaration are given below:

Incorrect:

**Ethics approval and consent to participate**

This study was approved by the Institutional Review Board of Eulji University (EU22-46, November 3, 2023).

Correct:

**Ethics approval and consent to participate**

This study was approved by the Institutional Review Board of Eulji University (EU23-46, November 3, 2023).
